# The Program for the Control of Visceral Leishmaniasis in Brazil: The Effect of the Systematic Euthanasia of Seropositive Dogs as a Single Control Action in Porteirinha, a Brazilian City with an Intense Transmission of Visceral Leishmaniasis

**DOI:** 10.3390/pathogens12081060

**Published:** 2023-08-18

**Authors:** João Carlos França-Silva, Rodolfo Cordeiro Giunchetti, Reysla Maria da Silveira Mariano, George Luiz Lins Machado-Coelho, Luciana de Almeida Silva Teixeira, Ricardo Andrade Barata, Érika Monteiro Michalsky, Marília Fonseca Rocha, Consuelo Latorre Fortes-Dias, Edelberto Santos Dias

**Affiliations:** 1 Institute of Biological Sciences, Federal University of Minas Gerais, Belo Horizonte 31270-901, MG, Brazil; franca@icb.ufmg.br (J.C.F.-S.); giunchetti@gmail.com (R.C.G.); reyslamariano@yahoo.com.br (R.M.d.S.M.); 2 Medicine School, Federal University of Ouro Preto, Ouro Preto 35402-163, MG, Brazil; gmcoelho1@gmail.com; 3 Department of Medical Clinics, Federal University of Triângulo Mineiro, Uberaba 38025-180, MG, Brazil; luciana.teixeira@uftm.edu.br; 4 Federal University of Vales do Jequitinhonha e Mucuri, Diamantina 39803-371, MG, Brazil; ricbarata@hotmail.com; 5 René Rachou Institute, Oswaldo Cruz Foundation, Belo Horizonte 30190-002, MG, Brazil; erika.monteiro@fiocruz.br; 6 Zoonoses Control Center, State University of Montes Claros, Montes Claros 39401-089, MG, Brazil; rfmarilia@hotmail.com; 7 Research and Development Center, Ezequiel Dias Foundation, Belo Horizonte 30510-010, MG, Brazil; consuelo.latorre@funed.mg.gov.br

**Keywords:** *Leishmania* (*Leishmania*) *infantum*, canine visceral leishmaniasis, human visceral leishmaniasis, *Lutzomyia longipalpis*, American visceral leishmaniasis, canine euthanasia

## Abstract

Background: Porteirinha is endemic for visceral leishmaniasis (VL), with intense disease transmission of the disease. We evaluated the impact of canine euthanasia as a single control measure on the incidence of VL in humans and canines. Methods: A prospective observational cohort study was carried out over four years (1998–2002) in 8 of the 12 neighborhoods of the city. The dynamics of canine visceral leishmaniasis (CVL) transmission were evaluated for 2 years, before beginning the screening–culling intervention. The comparative morbidity index (CMI) was used to stratify areas with the greatest risk of CVL, and the spatial distribution of human and canine VL cases was compared using univariate and bivariate K-functions. Results: Human cases conglomerated in three neighborhoods. Spatial clusters were detected for CVL in 1998, 2000, and 2001, but not in 1999, when greater spatial dispersion occurred. The screening and culling intervention reduced the number of human VL cases and decreased the incidence of CVL, mainly in neighborhoods with a high CMI. Conclusions: The systematic euthanasia of seropositive dogs was shown to be an effective control action of the Program for Control of Visceral Leishmaniasis (PCLV) in Brazil. The fundamental role of domestic dogs in the epidemiological chain of VL was reaffirmed.

## 1. Introduction

Visceral leishmaniasis (VL) is a serious public health and veterinary problem in Brazil [1,2,3], with territorial expansion that has reached all geographic regions of the country, including medium and large cities. The current epidemiological pattern differs from that initially constrained to the rural environment. The etiological agent *Leishmania (Leishmania) infantum* belongs to the Kinetoplastida order, Trypanosomatidae family, *Leishmania* genera (ROSS, 1903), and it is transmitted by the bite of female dipterous insects of the Psychodidae family belonging to the *Phlebotomus* and *Lutzomyia* genera in the Old and New World, respectively [4]. Ninety percent of human VL cases in South America are reported in Brazil. Epidemiological data from 1990 to 2009 show an average incidence of VL in humans of approximately 1.8 cases/100,000 habitants. Between 1994 and 2005, the average fatality rate was 5.5% per year. The high lethality rate remains the main problem in VL, and its occurrence has been linked to the presence of other comorbidities such as hepatic, renal, and cardiac diseases and HIV infection [3].

Among VL-endemic countries, Brazil is the only country that conducts a regular government program to monitor and prevent further expansion of the disease [5] through The Program for Control of Visceral Leishmaniasis (PCVL). The program comprises four basic public health measures: free referral for all reported human cases, euthanasia of seropositive domestic reservoirs (dogs), use of residual insecticides to control vector density (*Lu. longipalpis*), and rigorous epidemiological surveillance [1]. Subsequently, the Ministry of Health incorporated environmental management as a complementary recommendation for vector control and health education [6]. These actions have considerable operational and logistical costs and are complex and laborious in practice [7,8,9,10]. The efficiency is questionable due to low technical and scientific support concerning the fragilities related to serum diagnosis, low cost–benefit ratio of canine euthanasia of serum-positive dogs, and rapid replacement with new dogs by owners [9,10]. The success of PCVL was observed only when the control measures were carried out in an integrated manner [11,12,13].

The present study evaluated the impact and efficiency of the fast and systematic removal of seropositive dogs as an isolated VL control action in an endemic area of intense disease transmission in Brazil.

## 2. Materials and Methods

### 2.1. Study Area

Porteirinha is located in the northern region of the state of Minas Gerais (15°44′42″ S, 43°01′46″ W), 630 km from its capital, Belo Horizonte. The municipality occupies an area of 1788 km^2^ with an altitude of 567 m, within the “dry-land polygon”. The city comprises 12 neighborhoods distributed in hills in its higher region (São Judas Tadeu, Mato Verde, Vitória, and União) and wide openings or lower regions encompassing the Centro, Floresta, Morada do Parque, Ouro Branco, Renascença, São Sebastião, Kennedy, and Serranópolis neighborhoods (Figure S1). At the time of our study, the local population consisted of 35,465 inhabitants, with 41% living in the urban areas [14].

The geophysical characteristics of the municipality present a relief divided into three distinct parts: a high limestone hill (10%), a wavy section (50%), and a lower and flat region (40%) represented by the São Franciscan depression. The predominant phytogeography is the *cerrado*, with dense arboreal vegetation appearing in the humid parts of the valleys, especially on the banks of the main perennial (Gorutuba, Mosquito, Serra Branca, and Lages) and temporary (Mucambinho, Sítio Novo, Sanharol, and Cocos) rivers, all of which belong to the São Francisco River Basin. The climate is tropical and semi-humid, with an average temperature of 24 °C and a dry season lasting approximately 6 months per year. The rainy season extends from October to March, with an average annual rainfall of 600 mm [15].

### 2.2. Study Design

**Figure 1:**
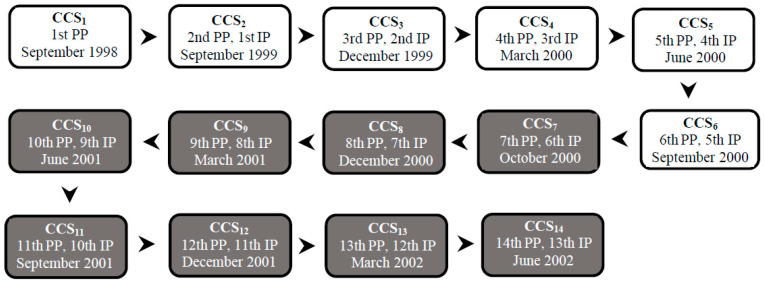
Flowchart of the canine census surveys (CCSs), prevalence points (PP), and incidence points (IP) of canine visceral leishmaniasis in Porteirinha, Minas Gerais State, Brazil. The CCSs were performed quarterly and are indicated by the starting month per quarter. Legend: white background—serological surveys for canine visceral leishmaniasis with no intervention; black background—serological surveys with systematic removal of seropositive dogs for euthanasia.

A prospective observational cohort was initiated in the September of 1998 through the first and only census survey (CCS) covering almost 100% of the canine population domiciled in Porteirinha. This study aimed to evaluate the epidemiological status of VL. This first stage was considered the study baseline and represented the first prevalence point (PP) in both urban and rural areas (Figure 1). The second CCS was performed in September 1999 and corresponded to the second PP and the first incidence point (IP). From this point until the end of the study, only the canine population domiciled in urban areas was followed. The zootechnical profile of the native canine population was compiled during CCS3. Although the PCVL comprises other control measures, none of them were applied during our four-year study, which allowed for the evaluation of the screening–culling action as a sole action in the period.

### 2.3. Serological Diagnosis of CVL by Immunofluorescence Antibody Test (IFAT)

Canine blood was collected by filter paper impregnation [16,17], and IFAT was performed as described previously [18]. Promastigotes from *Leishmania* (*Leishmania*) *mexicana* (strain MHOM/BR/1960/BH6) were used as antigens. Anti-dog IgG fluorescence-conjugated antibodies were produced by Biomanguinhos (FIOCRUZ, Brazil). Titers ≥1:40 were considered positive for CVL [19]. Seropositivity was confirmed by retesting a new sample collected via cephalic or jugular venipuncture [20]. Quality control was monitored by a reference laboratory (FUNED) of the National Network for Serodiagnosis of CVL.

### 2.4. Indicators of CVL Morbidity

The comparative morbidity index (CMI) was used to stratify areas with the greatest risk of CVL [21].

$$\mathrm{CMI}=\left(\mathrm{observed}\;\mathrm{no}.\;\mathrm{cases}/\mathrm{global}\;\mathrm{rate}\;\times \;\mathrm{area}\;\mathrm{population}\right)\times 100$$

where CMI values of 1 indicate medium risk, values >1 denote high risk, and values <1 indicate low risk. The incidence and prevalence rates were calculated as follows:

Incidence = (number of new cases of CVL/canine population exposed in the area) × 1000

Prevalence = (number of cases of CVL/canine population exposed in the area) × 100

### 2.5. Follow-Up of the Population Seropositive for CVL

In neighborhoods with great risk of VL transmission, the surviving seropositive dogs were maintained in their respective domiciles from CCS1 to CCS6 (Figure 1). In the two-year period, no intervention or control action was taken to avoid any interference with the local force of infection. The results of clinical examinations, suggestive clinical signs, and serological monitoring (serological titer) were recorded and transferred to a database. 

The active search and systematic removal of dogs seropositive for VL started in CCS7 and ended in CCS14 (Figure 1). 

### 2.6. Clinical and Serological Diagnosis of Human VL

VL diagnoses and treatment protocols were based on compatible clinical and laboratory findings [22]. Individuals with positive serology and clinical symptoms were considered symptomatic, and those with positive serology but no clinical symptoms were considered inapparent for VL.

### 2.7. Statistical Analysis

The database was constructed using ACCESS software version 8.0. Proportions were compared by chi-squared and Pearson correlation coefficient tests (*p* < 0.05). All households with cases of symptomatic or inapparent human VL between 1998 and 2001 or CVL were georeferenced using a GPS device (GARMIM-ETREX) and processed using MapInfo software version 6.0.

## 3. Results

During our four-year study, 40,387 indirect immunofluorescence reactions were performed, of which 556 samples yielded seropositive results for CVL, with an accumulated prevalence of 1.38% and accumulated incidence of 7.5 cases/1000 dogs/year.

In CCS1, 5071 dogs were examined (Table 1). Two hundred-ninety-one dogs (5.7%) were seropositive for the disease. Statistical comparison of the prevalence ratios in urban (4.4%) and rural (6.1%) areas indicated that rural dogs had a higher chance of contracting VL.

The dynamics of CVL transmission were evaluated for 2 years, from CCS1 to CCS6, before the beginning of the screening–culling action (Table 2). The number of dogs examined per survey varied from 1398 (CCS2) to 5071 (CCS1), in a total of 14,195 animals. The incidence rate ranged from 4.3 cases/1000/dogs/year in CCS7 to 15.9 cases/1000/dogs/year in CCS6.

Short-haired dogs had the highest chances of infection (Table S1). Most animals from the survey (75%, 1128 dogs) were mongrels, about 10% (150 dogs) belonged to 10 short-haired breeds (Chihuahua, Rottweiler, Weimaraner, Pinscher, American Pointer, Brazilian fila, Dachshund, Dobermann, Dog German, and Boxer), and 15% (226 dogs) were long-haired from six breeds (Poodle, Pekingese, Akita, Siberian Husky, German Shepherd, and Cocker). Among mongrel dogs, the average CVL seropositivity was 2.9%. The most affected breeds were Dobermann (22.2%) and Brazilian Fila (2.6%) (data not shown). The seropositivity rates for males (892 dogs) and females (612 dogs) did not differ significantly. Therefore, both sexes had the same probability of Leishmania infection.

In the CCS3, CVL indicators were calculated for each neighborhood (Table S2). CVL was detected in 8 of the 12 neighborhoods of Porteirinha. Twenty-three new cases of CVL were diagnosed during the two-year study period, with an average incidence of 15.2 cases/1000 dogs/year. The CVL incidence varied from 8.8 in Vila Vitória to 40.0 in Vila União. Six neighborhoods did not introduce new dogs, and the CVL incidence remained at zero. The CVL prevalence varied from 0 to 4.38 cases. Five of the twelve neighborhoods had a CMI >1, indicating a high risk of CVL transmission. 

In CCS4, at the end of the rainy season, we observed the highest rates of survival for seropositive dogs (86.6%), growth of the seropositive canine population (24.9%), seropositivity (3.0%), incidence (15.9 cases/1000/dogs/year), and number of new cases (30), and the lowest mortality rate (13.4%) for seropositive dogs. During follow-up, we observed several canine deaths and dogs missing from their former homes, suggesting an important migration rate of seropositive animals. 

Almost all neighborhoods reported CVL cases between September 1998 and September 2001, except for Morada do Parque (Table 3). 

**Figure 2:**
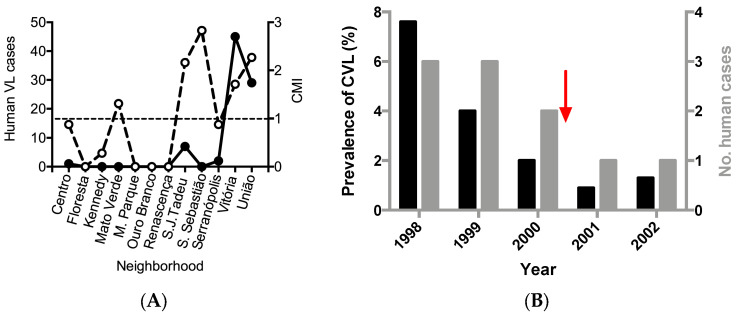
(**A**) Number of human visceral leishmaniasis cases and comparative morbidity index (CMI) for canine visceral leishmaniasis, per neighborhood of Porteirinha (Minas Gerais state, Brazil) from 1998 to 2001. Legend: black dots—human cases (symptomatic and inapparent); white dots—CMI. CMI values above 1 (dashed line) indicate areas with higher risk for CVL. (**B**) Effect of the systematic withdrawal of seropositive dogs for canine visceral leishmaniasis in the number of human cases of visceral leishmaniasis in União neighborhood from 1998 to 2002. Porteirinha, Minas Gerais State, Brazil. Prevalences are in black bars and number of human cases are in grey bars. The red arrow indicates the beginning of the systematic screening–culling intervention.

Floresta and Mato Verde had the lowest numbers of cases (two each), whereas the largest number occurred in São Judas Tadeu (54 cases). During the same period, 84 human cases of VL (symptomatic or inapparent) were reported. The largest number of households with positive VL serology (45) was in Vitória, of which 42 were asymptomatic. In contrast, of the 29 patients in Vila União, 11 were symptomatic. Human cases occurred in neighborhoods with CMI >1, except for one single case in Centro (CMI = 0.88) (Figure 2A). Three of the five high-risk neighborhoods registered human cases of VL. Among the 19 patients with symptomatic VL from all neighborhoods, 11 lived União. Because União had CMI = 1.71 and 58% of the symptomatic cases of VL, we used this neighborhood as our model for the analysis of the screening–culling intervention. Notably, the incidence rate of human VL cases decreased by from eight to two cases after the intervention (Figure 2B). A concomitant reduction in the prevalence of CVL was noted.

**Figure 3:**
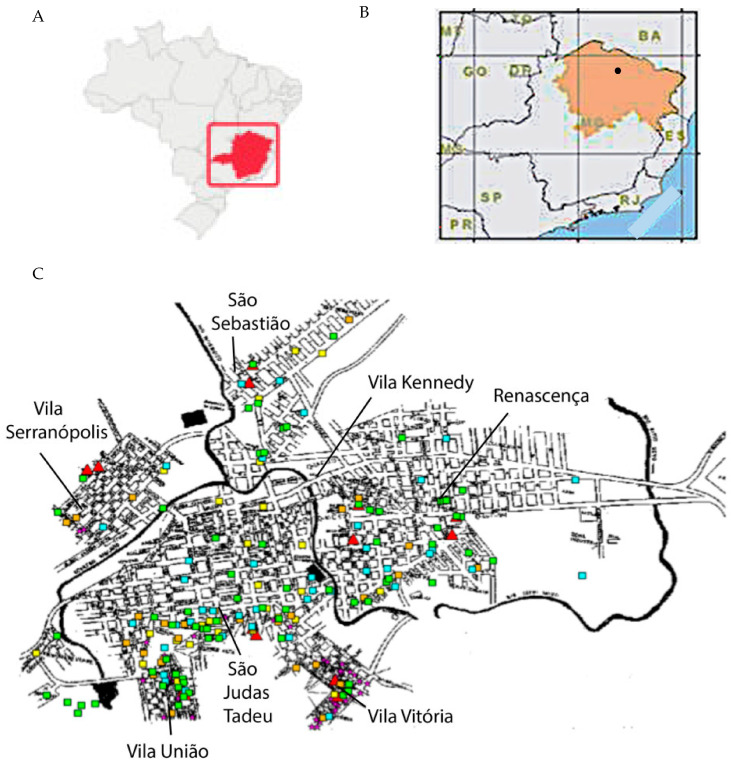
Study area. (**A**). Geographical location of Minas Gerais State (in red) in Brazil. (**B**). Dry land polygon in Minas Gerais State (in orange). The black circle indicates the location of Porteirinha. (**C**). Geopolitical map of Porteirinha with the spatial distribution of human and canine cases of visceral leishmaniasis between 1998 and 2001. Human cases are marked with purple stars. Canine cases are indicated by colored rectangles per year: orange—1998; yellow—1999; green—2000; blue—2001. Red triangles refer to sites of entomological captures (not addressed here). São Judas Tadeu, União, and Vitória are located in the higher part of the city.

The spatial distribution of human and canine cases is shown in a geopolitical map of Porteirinha (Figure 3). Kernel maps were evaluated using the respective K (univariate)-function for the annual georeferencing of the spatial distribution of households with human and canine VL cases. The univariate K-function for each year of the spatial distribution of CVL cases was significant for 1998, 2000, and 2001, when spatial clusters were observed. K-function was not significant in 1999, when greater dispersion occurred in other regions of the city.

**Figure 4:**
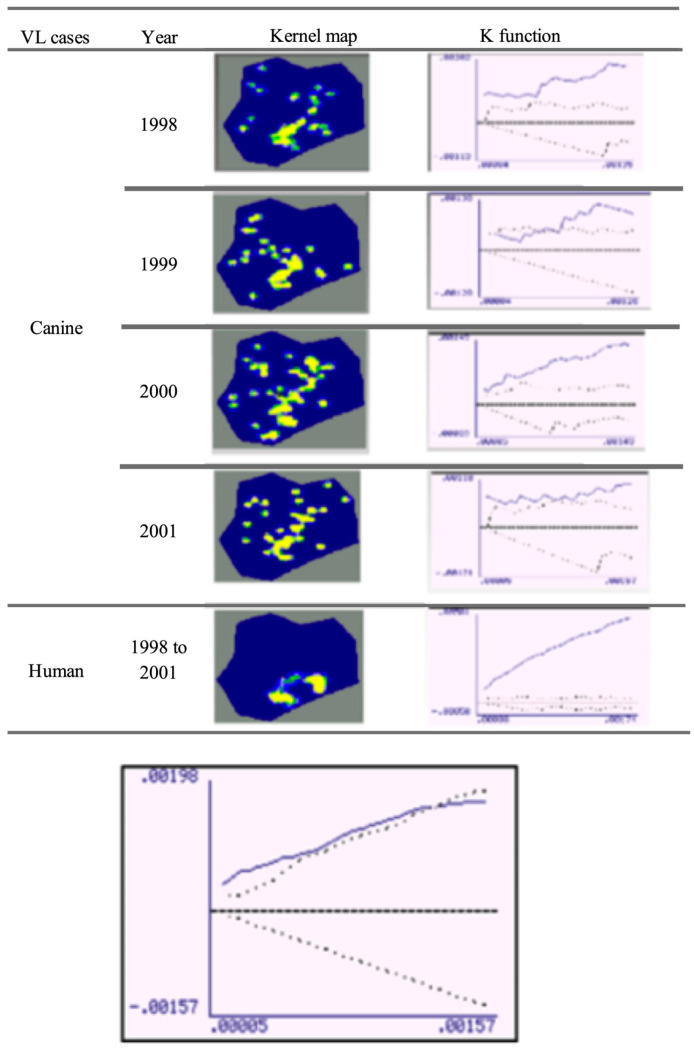
Kernel map and respective uni-variated K-function (top) and bi-variated K-function for the spatial distribution of human and canine cases of visceral leishmaniases. Period: 1998 to 2001. Porteirinha, Minas Gerais State, Brazil.

The univariate K-function for the spatial distribution of human VL cases (symptomatic and inapparent) was highly significant, confirming the existence of a restricted conglomerate in the neighborhoods of São Judas Tadeu, Vitória, and União. Figure 4 shows the bivariate K-function for the spatial distribution of human and canine cases, of which, dispersion patterns differed significantly. CVL was dispersed throughout the city, and human VL was mostly concentrated in São Judas Tadeu, Vitória, and União.

## 4. Discussion

In Brazil, the CVL seropositivity in endemic areas ranges from 5% to 35% [15,17,23,24]. In Porteirinha, the CVL seropositivity (5.7%) was close to the lowest value in the range. Dogs in rural areas were more prone to contracting CVL than those living in urban areas. In urban areas, short-haired dogs had a higher risk of contracting CVL. Mongrel dogs (2.9%) and Fila Brasileira (2.6%) showed similar seropositivity rates, which differed from that of Dobermanns (22.2%), the most affected breed. Dobermann was also the most affected breed, with 35.3% seropositivity, in the Lisbon metropolitan region (Portugal). Sideris et al. [24] reported that this short-furred animal in Athens (Greece) was the most susceptible to CVL and was more easily bitten by sandflies. Boxers, together with German Shepherds, were also the most affected breeds in a study conducted in France [25]. The authors suggested that the high susceptibility was because both are working breeds, mostly acting as guard dogs; therefore, they are more exposed to Leishmania infection. In the context of *Leishmania* infection, dogs are distinguished more by their occupation than by their place of residence [26,27]. Working dog breeds in particular experience a greater force of infection compared to companion dogs. They usually sleep outside and are often exposed to sandfly bites. Companion dogs that generally stay inside their owners’ homes are less exposed to bites. In addition, they are more often taken to veterinary clinics for grooming and bathing, thus being exposed to products that drive flies away.

In the present study, male and female dogs were equally likely to show CVL seropositivity. Similar results were reported by others in Montes Claros and in different countries [17,24,28,29]. In contrast, higher seropositivity rates were reported in male dogs in France [30].

In our study, IFAT was used as a screening and confirmatory test; therefore, we cannot rule out possible cross-reactions with other trypanosomatids, since the IFAT reaction does not distinguish infections, for example, between *Le.* (*Le.*) *infantum*, *Leishmania (Leishmania) braziliensis* and even *Trypanosoma cruzi* [19].

Brazil has experienced a clear territorial expansion and a significant increase in the number of human VL cases [1,2,3]. The epidemic outbreaks recorded in important urban centers in Brazil demonstrated how the migration from the countryside to large cities influenced changes in the epidemiological profile of VL. In the last three decades, many human cases were reported in several Brazilian capitals [1,3,31,32,33,34].

The factors affecting the epidemiology of VL in Porteirinha may be the same as those observed by several authors [34,35,36,37,38], including poverty; malnutrition; large numbers of infected dogs; high vector density in households and peridomiciles; large numbers of domestic animals; poor sanitary conditions; low socioeconomic indices; and, possibly, the differentiated ecological valence of the species *Lu. longipalpis*.

Kernel maps for human and canine VL in Belo Horizonte showed a significant correlation between the occurrence of human and canine VL [38]. In Porteirinha, the pattern of dispersion of human VL and CVL were different, remaining restricted to the São Judas Tadeu, Vitória, and União, which are subnormal conglomerates without basic sanitary infrastructure, located in the foothills of the city. In these places, where a population of low socioeconomic status resides, high densities of *Lu. longipalpis* have been observed, along with numerous domestic animals raised in chicken coops, pigsties, and corrals, living with humans, as well as a high number of domiciled dogs [39,40].

In the present study, the existence of a well-defined spatial cluster of canine cases that spatially coincided with human cases was significant when evaluated using the bivariate K-function. While CVL was dispersed, human VL was restricted to the upper parts of the city, clearly suggesting the association of three main components (the presence of seropositive dogs, association with the presence of susceptible humans, and high vector density) as determining factors involved in the eco-epidemiological chain. The Kernel maps showed a correlation between areas with active transmission of human infection and those with a significant prevalence of canine infection. The identification of areas with a greater risk of transmission is important, as it not only evidenced the local urbanization of VL but is also useful in directing entomological studies and subsidizing protocols for integrated control actions in prioritized areas.

In general, euthanasia of seropositive dogs by PCVL takes an average of 120 days, enough time for transmission to other susceptible dogs and humans, justifying, in part, why the efficiency of the intervention has been questioned. Herein, seropositive dogs were excluded 30 days after serodiagnosis. In our model neighborhood (União), the intervention significantly reduced CVL prevalence and remained stable throughout the study period, always remaining <2.0% from the CCS_7_ onwards. The number of human cases notably decreased by 75%, mainly after the intervention, suggesting that the systematic removal of seropositive dogs could be more efficient when adopted faster in endemic areas, especially when the force of infection is characterized by a high rate of transmission of human cases. The chi-squared value for human cases in relation to the population residing in the União corresponded to a probability of error of 5–10%. The low number of human cases reported in the study period may have caused bias and prevented it from reaching significance (*p* < 0.005).

According to Costa et al. [9], no high-evidence publications have established an association between the occurrence of human VL cases and the seroprevalence of CVL or the removal of infected dogs. Our results, as well as those reported by Ashford et al. [41], demonstrate that the systematic removal of the seropositive canine population may be insufficient as a measure to control VL, but it could reduce the strength of infection among dogs and temporarily affect the cumulative incidence of seroconversion among them. Canine control alone may not be an effective methodology to reduce the number of infectious dogs or the consequent incidence of human diseases, but the use of a highly sensitive diagnostic method, as well as a reduction in the time between serodiagnosis and the consequent euthanasia of seropositive dogs, may lead to a significant reduction in the incidence of canine and human VL [10,42]. Five years of canine control intervention reduced the CLV seroprevalence in Ceará (Brazil) to between 0.5% and 1% [43]. In a case–control study conducted in the same state, a significant decrease in the incidence rate of human disease was observed in areas where seropositive dogs were euthanized [44].

In 2011, the Ministry of Health modified the serodiagnosis protocol for CVL by incorporating the dual-path platform chromatographic immunoassay (DPP^®^ Bio-Manguinhos-FIOCRUZ-RJ) as a screening test and enzyme-linked immunosorbent assay (ELISA) as a confirmatory test [6], considerably speeding up testing. The screening–culling control measure is badly accepted by the population, for obvious reasons. Even so, until now, every dog presenting positive results in the current diagnosis tests is considered *Leishmania* infected and recommended for euthanasia [45]. The effectiveness of the control measure is controversial in the literature. There are reports of significant reductions in canine and human VL cases after the removal of seropositive dogs, as well as no effect after massive euthanasia [41,42,43,44,45,46,47,48,49]. An aggravating factor is that clinically asymptomatic dogs, and infected dogs with divergent serological results are maintained in people’s homes, where they act as potential sources for *Leishmania* vector infection and facilitate the disease transmission cycle [50,51,52,53]. Recently, the Brazilian Ministry of Health approved a proposal for the incorporation of collars impregnated with insecticide (4% deltamethrin) for the control of visceral leishmaniasis in priority municipalities [54]. 

Although 20 years have passed since the development of the present study, it remains a model for the evaluation of PCLV in endemic areas of intense VL transmission in Brazil. It is among the first to apply geoprocessing to the spatial epidemiology of VL in Brazil. Kernel maps as well as the K-function clearly showed a positive and significant correlation between the occurrence of human and canine cases. An important point is that the screening–culling was the only control measure applied during our study. In addition, the same epidemiological setting (Porteirinha) was analyzed and compared, in contrast to other epidemiological studies that compare areas with different degrees of VL transmission and may introduce artifacts and bias the results.

## Figures and Tables

**Table 1:** Comparison of seropositivity for canine leishmaniasis registered in the first canine census survey (CCS1) and first prevalence point (1st PP) in urban and rural areas of Porteirinha, Minas Gerais State, Brazil.

Area	No. Dogs		Prevalence (%)	Confidence Interval (95%)
	Examined	Positive		
Urban	1230	55	4.4	3.39~5.78
Rural	3841	236	6.1	5.41~6.96
Total	5071	291	-	-
Mean	-	-	5.7	5.11~6.41

Odds ratio 1.37 (1.01~1.88); Yates corrected c^2^ = 4.04, *p* = 0.044.

**Table 2:** Data from the canine census surveys (CCSs) between 1999 and 2000, before the canine control action in the urban area of Porteirinha (Minas Gerais, Brazil).

Indicators	Canine Census Surveys				
	CCS_2_	CCS_3_	CCS_4_	CCS_5_	CCS_6_
No. dogs examined	1398	1504	1878	2045	2299
Accumulated no. of dogs examined	1398	2902	4780	6825	9124
No. seropositive dogs (new cases)	14	23	30	12	10
Accumulated no. of seropositive dogs	14	37	67	79	89
No. seropositive dogs (followed up)	14	30	56	51	41
No. seropositive dogs (surviving)	14	7	26	39	31
Survival rate (%)	100	50	86.6	69.6	60.8
Mortality index (%)	0	50	13.4	30.4	39.2
Growth rate of seropositive	-	7.6	24.9	8.9	11
canine population (%)					
Average prevalence rate (%)	1	2.5	3	2.5	1.8
Average incidence rate (cases/1000 dogs/year)	10	15.3	15.9	5.9	4.3

**Table 3:** Distributions per year of georeferenced domiciles with canine and human cases of visceral leishmaniasis from 1998 to 2001. Porteirinha, Minas Gerais State, Brazil.

Neighborhood	Canine Cases per Year				Total	Human Cases		Total
	1998	1999	2000	2001		(from 1998 to 2001)		
						Symptomatic	Inapparent	
Centro	0	6	6	3	15	1	0	1
Floresta	0	0	1	1	2	0	0	0
Mato Verde	0	1	1	0	2	0	0	0
Morada do Parque	0	0	0	0	0	0	0	0
Kennedy	5	1	9	5	20	0	0	0
Ouro Branco	1	0	2	3	6	0	0	0
Renascença	3	0	11	7	21	0	0	0
São Judas Tadeu	18	11	12	13	54	4	3	7
São Sebastião	3	5	8	4	20	0	0	0
Serranópolis	3	3	3	2	11	0	2	2
Vitória	6	5	4	2	17	3	42	45
União	5	5	14	3	27	11	18	29
Total	44	37	71	43	195	19	65	84

## Data Availability

Data supporting the reported results are available upon request.

## Supplementary Materials

The following supporting information can be downloaded at: https://www.mdpi.com/article/10.3390/pathogens12081060/s1, Supplement S1: Figure S1: Partial views of the city of Porteirinha, Minas Gerais state, Brazil. A. Higher region. B. Supplement S2: Table S1: Relative distribution of coat type and sex associated with the prevalence ratio evaluated in the third canine census survey (CCS3). Porteirinha, Minas Gerais state, Brazil. Supplement S3: Data from the 3rd canine census survey (CCS3) per neighborhood of the urban area of Porteirinha, Minas Gerais state, Brazil. Incidence is expressed by the number of new cases of CVL/canine population exposed in the area ×1000. Prevalence is expressed by the number of cases of CVL/canine population exposed in the area ×1000. CMI stands for Comparative Morbidity Index.
