# Supplementary material for: The Program for the Control of Visceral Leishmaniasis in Brazil: The Effect of the Systematic Euthanasia of Seropositive Dogs as a Single Control Action in Porteirinha, a Brazilian City with an Intense Transmission of Visceral Leishmaniasis
Source: Pathogens. 2023 Aug 18;12(8):1060. doi: 10.3390/pathogens12081060 (PMC10460051; doi:10.3390/pathogens12081060)
Supplement: Supplementary file 1 [file pathogens-12-01060-s001.zip › pathogens-2527634-supplementary.pdf]

Supplement S1

Figure S1. Partial views of the city of Porteirinha, Minas Gerais state, Brazil. A. Higher region. B. Lower region

A

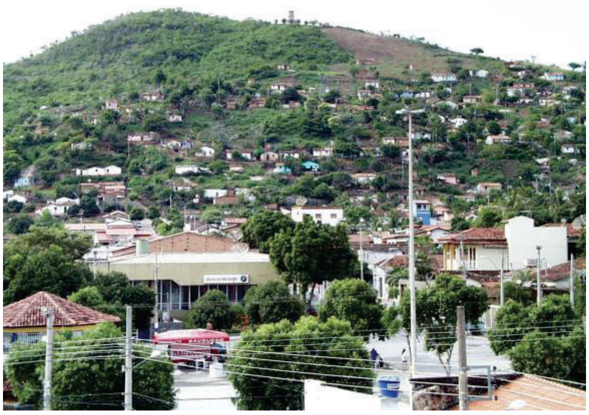

B

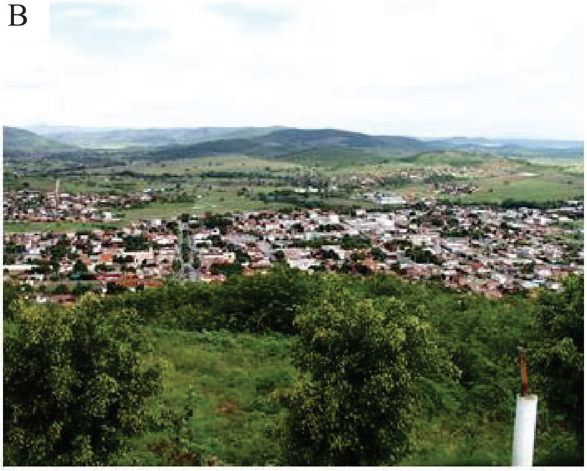

Supplement S2

Table S1. Relative distribution of coat type and sex associated with the prevalence ratio evaluated in the third canine census survey (CCS_3). Porteirinha, Minas Gerais state, Brazil.

Characteristics		Relative distribution (%)	No. dogs		Prevalence and confidence interval (%)
			Examined	Positive	
Coat type	Long-haired	18.2	274	2	0.7 (0.09 2.59)
	Short-haired	81.8	1230	35	2.8 (1.93 3.83)
	Total	100.0	1504	37	2.46 (1.76 3.41)
	Odds ratio= 3.98 (0.93 24.09); relative risk 1.02 (1.01 1.04); c^2 (Pearson) 4.18, p = 0.0409				
Gender	Female	40.7	612	16	2.61 (1.55 4.30)
	Male	59.3	892	21	2.35 (1.50 3.63)
	Total	100.0	1504	37	2.46 (1.76 3.41)
	Odds ratio 1.1 (0.58 2.15) ; Yates corrigido c^2 = 0.02, p = 0.880				

Supplement S3

Table S2. Data from the 3rd canine census survey (CCS_3) per neighborhood of the urban area of Porteirinha, Minas Gerais state, Brazil. Incidence is expressed by the number of new cases of CVL/canine population exposed in the area x 1000. Prevalence is expressed by the number of cases of CVL/canine population exposed in the area x 1000. CMI stands for Comparative Morbidity Index.

Neighborhood	No. dogs		Seropositive dogs			Incidence	Prevalence	CMI
	New	Introduced	New	Existent	Total			
Centro	21	38	5	6	320	15.6	1.87	0.88
Floresta	5	9	0	0	43	0	0	0
Morada do Parque	0	3	0	0	8	0	0	0
Ouro Branco	12	24	0	0	144	0	0	0
Renascença	8	17	0	0	124	0	0	0
São Judas Tadeu	10	49	9	12	237	38	5.06	2.16
São Sebastião	5	19	0	5	97	0	5.15	2.83
Vila Kennedy	11	35	0	1	149	0	0.67	0.28
Vila Mato Verde	5	8	1	1	32	31.3	3.12	1.31
Vila Serranópolis	10	15	3	3	136	22.1	2.2	0.88
Vila União	5	15	4	4	100	40	4	1.71
Vila Vitória	13	12	1	5	114	8.8	4.38	2.27
Total	105	244	23	37	1504	15.2	2.46	-
